# First report of plasmid-mediated colistin resistance *mcr-8.1* gene from a clinical *Klebsiella pneumoniae* isolate from Lebanon

**DOI:** 10.1186/s13756-020-00759-w

**Published:** 2020-06-26

**Authors:** Tamara Salloum, Balig Panossian, Ibrahim Bitar, Jaroslav Hrabak, George F. Araj, Sima Tokajian

**Affiliations:** 1 Department of Natural Sciences, School of Arts and Sciences, Lebanese American University, Byblos Campus, P.O. Box 36, Byblos, Lebanon; 2 Department of Microbiology, Faculty of Medicine, University Hospital Pilsen, Charles University, Pilsen, Czech Republic; 3 Biomedical Centre, Faculty of Medicine, Charles University, Pilsen, Czech Republic; 4 Department of Pathology and Laboratory Medicine, American University of Beirut Medical Center, Beirut, Lebanon

**Keywords:** Colistin, *mcr-8*, Hybrid assembly, Plasmid, *Klebsiella pneumoniae*, IncFIA, IncR, IncHI1B

## Abstract

Colistin is considered as a last resort treatment for infections caused by multidrug-resistant Enterobacteriaceae. Plasmid-mediated mobile colistin resistance (*mcr*) genes contributed to the global spread of colistin resistance. This is the first report of plasmid-mediated colistin resistance *mcr-8* gene from a clinical *Klebsiella pneumoniae* K9 isolate recovered from Lebanon. The isolate was characterized phenotypically and genotypically through both short and long read whole-genome sequencing, plasmid typing and conjugation assays. k9 belonged to sequence type 15 and harbored 31 antimicrobial resistance genes. The *mcr-8.1* variant was carried on a novel ~ 300 kb multireplicon plasmid having IncFIA, IncR and IncHI1B. The plasmid was conjugative and carried a plethora of antimicrobial resistance determinants. The introduction of novel *mcr* variants in Lebanon poses an alarming health concern. Surveillance and screening for colistin resistant Enterobacteriaceae and *mcr* in livestock, animal farms, imported meat and poultry is highly recommended along with monitoring antibiotic use.

## Introduction

Colistin is the last-line treatment option for infections caused by carbapenem resistant Enterobacteriaceae such as carbapenem-resistant *Klebsiella pneumoniae* (CRKP) [1]. The first plasmid mediated colistin resistance gene, *mcr-1*, was identified in 2015 in Enterobacteriaceae, mainly *Escherichia coli* and *K. pneumoniae,* collected from animals and humans in China [2]. To date, nine different plasmid-mediated *mcr* variants have been described, designated as *mcr-1 -9*, isolated from humans, animals and the environment [3].

*mcr-8* located on an IncFII-type conjugative plasmid was first described in 2018 in China in *K. pneumoniae* collected from both animals and humans [4]. Shortly after, it was detected in Laos [5] and Algeria [6].

The first report of a colistin resistant *K. pneumoniae* in Lebanon was in 2017 [7]. *mcr-1*-positive *E. coli* from a human clinical isolate in Lebanon has been recently reported [8]. On the other hand, *mcr-1* was widely detected in Gram-negative bacilli isolated from poultry [9], swine farms [9] and water sources in Syrian refugee camps [10]. This is accordingly, the first report of the complete sequence of a novel multireplicon conjugative plasmid carrying colistin resistance *mcr-8.1* gene variant in an ST-15 *K. pneumoniae* isolate recovered from Lebanon.

## Materials and methods

### Bacterial isolate

The isolate was recovered from the urine sample of a 50-years old female patient on the 28th of August 2018 at the Clinical Microbiology Laboratory of the American University of Beirut Medical Centre (AUBMC), Lebanon. The identification of the isolate was performed using Matrix-Assisted Laser Desorption/Ionization Time of Flight (MALDI-TOF) system (Bruker Daltonik, GmbH, Bremen, Germany). The isolate was identified as *Klebsiella pneumoniae* and designated as k9.

### Antimicrobial susceptibility testing

Antimicrobial susceptibility testing was performed using the disk diffusion assay on Mueller-Hinton agar and included a panel of 24 antibiotics belonging to 16 different classes (Table S1). The obtained data was interpreted according to the guidelines of the clinical & laboratory standards institute (CLSI) [11]. The minimal inhibitory concentrations (MICs) of ertapenem, imipenem and meropenem were determined using the E-test methodology (AB BIODISK, Solna, Sweden) according to the manufacturer’s guidelines. MIC of colistin in k9 and in the colistin resistant *E. coli* transconjugant was further determined by using the broth microdilution method according to the recommendations from the European Committee on Antimicrobial Susceptibility Testing (EUCAST) [12].

### DNA extraction

DNA extraction was performed from fresh bacterial colonies using the Nucleospin® Tissue kit (Macherey-Nagel, Germany) according to the manufacturer’s instructions.

### *mcr-8* PCR

The presence of *mcr-8* was confirmed through PCR using MCR-8F 5′-AACCGCCAGAGCACAGAATT-3′ and MCR-8R 5′-TTCCCCCAGCGATTCTCCAT-3’primer pair as previously described [4].

### Plasmid replicon typing

Plasmid characterization was performed using the DIATHEVA plasmid based replicon typing (PBRT) kit (Diatheva, Fano, Italy) through a polymerase chain reaction (PCR) based replicon typing method consisting of eight multiplex PCR assays for the amplification of 25 replicons: A/C, B/O, FIA, FIB, FIB-M, FIC, FII, FIIK, FIIS, HI1, HI2, HIB-M, I1, I2, K, L/M, N, P, R, T, U, W, X1, X2, and Y found in the family Enterobacteriaceae. Positive controls were included for all reactions. All PCR reactions were performed according to the manufacturer’s instructions.

### Conjugation assay

Conjugation was performed with an azide-resistant *E. coli* J53 strain. Selection of transconjuguants was done on Uriselect agar (Bio-Rad, France) supplemented with 100 mg/L sodium azide and 4 μg/ml colistin. *E. coli* transconjugants were typed by PBRT to detect plasmid replicons.

### Whole-genome sequencing

Genomic libraries were constructed using the Nextera XT DNA library preparation kit with dual indexing (Illumina). The libraries were sequenced on an Illumina MiSeq with 250 bp × 2 read length. Genome assembly was performed de novo using Spades Genome Assembler Version 3.6.0 [13]. Quality control check was performed using FastQC version 0.11.5 [14].

The *mcr8-*carrying plasmid was sequenced using PacBio long-read sequencing technology on the Sequel platform (Pacific Biosciences, CA, USA). Library preparation was performed according to the manufacturer’s instructions for microbial multiplexing. G-tubes (Covaris, USA) were used for DNA shearing, and no size selection was performed.

### Genome analysis

The assembled genomes were annotated using the RAST online server (http://rast.nmpdr.org) [15]. MLST 2.0 [16], ResFinder 3.0 [17], PointFinder 3.1.0 [18] and Plasmid Finder 2.1 [19] available on the Centre for Genomic Epidemiology website (www.genomicepidemiology.org) were used to determine the ST, presence of resistance genes and plasmid content, respectively. The sequences encoding for plasmids were then extracted and aligned to references obtained from NCBI as previously described [20]. The ISfinder database (https://www-is.biotoul.fr) [21] was used to identify insertion sequences. In silico capsular typing of the *wzi* gene was performed using Kaptive (www.kaptive.holtlab.net) [22]. The presence of putative virulence factors was screened using the VF scheme available on http://bigsdb.pasteur.fr. PlasmidsSpades was used to assemble the plasmid sequences [23]. Plasmid sequences were extracted and aligned with corresponding reference strains using BioNumerics software version 7.6.1 (Applied Maths, St-Martens-Latem, Belgium). Blast Ring Image Generator (BRIG) version 0.95 was used to visualize the plasmid comparisons [24].

The presence of known mutations conferring colistin resistance on the *pmrA*, *pmrB*, *pmrC*, *pmrD*, *phoP*, *phoQ*, *mgrB*, *crrA* and *crrB* genes were investigated in silico using both nucleic and amino acid alignments compared to the wild-type sequence of *K. pneumoniae* MGH 78578 (GenBank accession no. CP000647). SNAP [25] (http://www.hiv.lanl.gov) was used to calculate synonymous and non-synonymous substitution rates based on the codon-aligned nucleotide sequences of *pmrA* and *pmrB* against the *K. pneumoniae* MGH 78578 references.

## Results

### Genome content

K9 genome size was 5,656,813 bp with a GC content of 56.9%. The best match for the capsular locus was KL107 (Blastn identity: 89.60%) with *wzc* 931 and *wzi* 178 allelic types. Four Inc. groups were detected in k9 using PlasmidFinder [19] including IncFIA (HI1) (Accession no. AF250878), IncFIB (pKPHS1) (Accession no. CP003223), IncHI1B (Accession no. JN420336) and IncR (Accession no. DQ449578). The presence of the four replicons was also confirmed by PBRT. k9 also harboured virulence genes encoding for iron uptake (*kfuA* and *kfuC*) and fimbriae (*mrkB-D*, *mrkH-J*).

k9 was compared to all other *mcr-8.1* positive isolates recovered so far (Table 1). These included pKP91 (Accession no. MG736312) [4], pKP95 (Accession no. VDEL010000) [6], *R. ornithinolytica* QDRO2 (Accession no. MK097469.1) [26] KP91, KP95 and QDRO2 were obtained from swine fecal material in China [4], a clinical oral cavity pus sample in Algeria [6], and chicken cloaca in China [26], respectively (Table 1). k9 harboured significantly more resistant determinants (*n* = 31) than other *mcr-8.1* positive isolates with 16, 18 and 23 antimicrobial resistance genes (AMRs) detected in isolates KP95, KP91 and QDRO2, respectively. The latter isolates represented different STs and were recovered from both humans and animal sources [4, 6, 26].

**Table 1:** Characteristics of all reported *mcr-8.1*carrying isolates. COL: colistin; MIC: minimal inhibitory concentration; CC: clonal complex

#	Isolate	Source	City, Country	COL MIC (mg/L)	Antibiotic Resistance Genes	ST	CC	Reference
**1**	*K. pneumoniae* k9	Urine (Human)	Beirut, Lebanon	10	*mcr-8.1, bla*_DHA-1_*, bla*_SHV-12_*, bla*_SHV-13_*, bla*_SHV-31_*, bla*_SHV-86_*, bla*_SHV-129_*, bla*_SHV-155_*, bla*_SHV-172_*, aac(6′)-Ib-cr, aadA1, aadA16, aadA2b, aph(3′)-Ia, aac(6′)-Ib-cr, oqxA, oqxB, qnrB2, qnrB4, qnrB52, sul1, sul3, fosA, mph(A), mph(E), msr(E), tetD, dfrA27, cmlA1, floR, arr-3*	15	23	This study
**2**	*K. pneumoniae* KP91	Swine feces (Animal)	Shandong, China	16	*mcr-8.1, bla*_CTX-M-14_*, bla*_SHV-1_*, strA, strB, armA, aph(4)-Ia, mph(E), msr(E), oqxA,qnrB4, sul1, sul2, sul3, tet(A), tet(B), tet(34), dfrA12*	42		[4]
**3**	*K. pneumoniae* KP95	Oral cavity pus (Human)	Sétif, Algeria	8	*mcr-8.1, bla*_OXA-48_*, bla*_CTX-M-15_*, bla*_SHV-94_*,bla*_OXA-1_*, bla*_TEM-1B_*, aph(3′)-la, aac(6′)-lb-cr, aph(3″)-lb, aph(6)-ld, oqxB,oqxA, fosA, catB3, sul2, dfrA14*	336		[6]
**4**	*R. ornithinolytica* QDRO2	Chicken cloaca (Animal)	Shandong, China	8	*mcr-8.1, bla*_TEM-1B_*, bla*_OXA-1_*, bla*_SHV-73_*, aac(3)-IVa, aph(4)-Ia, aadA2, fosA, mph(A), mph(E), cat, floR, cml, qnrS4, oqxAB, nrB52, sul1, sul2, sul3, tet(A), tet(34), tet(O), tet(B)*			[26]

### Antimicrobial susceptibility testing

k9 had a colistin MIC of 10 μg/mL (Table S1). It was resistant to 20 of the tested antibiotics, showed intermediate resistance to cefepime, and remained susceptible to meropenem, imipenem, ertapenem, and fosfomycin and was accordingly classified as being extensively drug resistant (XDR) [27].

### Resistance genes

Studying antibiotic resistance mechanisms revealed the presence of 31 different resistance genes on the plasmid including resistance to: β-lactams (*bla*_DHA-1_, *bla*_SHV-12_, *bla*_SHV-13_, *bla*_SHV-31_, *bla*_SHV-86_, *bla*_SHV-129_, *bla*_SHV-155_, and *bla*_SHV-172_), aminoglycosides (*aac(6′)-Ib-cr, aadA1, aadA16, aadA2b* and *aph(3′)-Ia*), quinolones (*aac(6′)-Ib-cr*, *oqxA*, *oqxB*, *qnrB2*, *qnrB4*, and *qnrB52*), sulphonamides (*sul1* and *sul3*), fosfomycin (*fosA*), macrolides (*mph(A),mph(E),msr(E)*), tetracycline (*tetD*), trimethoprim (*dfrA27*), phenicol (*cmlA1*, *floR*), rifampicin (*arr-3)* and colistin (*mcr-8*).

The presence of known mutations conferring colistin resistance were also investigated. *pmrD*, *phoP*, *phoQ* and *mgrB* showed 100% identity when compared to the wild-type reference strain *K. pneumoniae* MGH 78578, while *crrA* and *crrB* were not detected. *pmrA* and *pmrB* however, showed 40.1 and 49.1% similarity, respectively, to the wild-type reference strain. The rates of synonymous and non-synonymous substitutions were further investigated. SNAP (http://www.hiv.lanl.gov) revealed a dN/dS ratio of 1.131 for *pmrA* and 1.181 for *pmrB* indicating a positive selection for non-synonymous mutations (Figure S1).

Mutations in *ompK*, an outer membrane porin, were investigated using a codon-aligned nucleotide sequences of an intact *ompK35* (Accession no. AJ011501), *ompK36* sequence (Accession no. Z33506.1) and *ompK37* (Accession no. AJ011502.1). We detected a premature stop codon in *ompK35* (Y76*), and 22 different amino acids in *ompK36* and two in *ompK37*. A premature stop codon was also detected in *ramR* (K194*); a positive regulator of the AcrAB efflux system, compared to an intact *ramR* reference genes (Accession no. KY465996.1).

### *mcr-8* genetic environment

Blast analysis revealed that the *mcr* variant was *mcr-8.1* showing 100% identity to *mcr-8.1* carried by *K. pneumoniae* plasmid pKP91 (Accession no. MG736312), a ~ 90-kb IncFII-type plasmid obtained from swine fecal material in China [4] and in *Raoutella ornithinolytica* QDRO2 (Accession no. MK097469.1) [26]

**Fig. 1:**
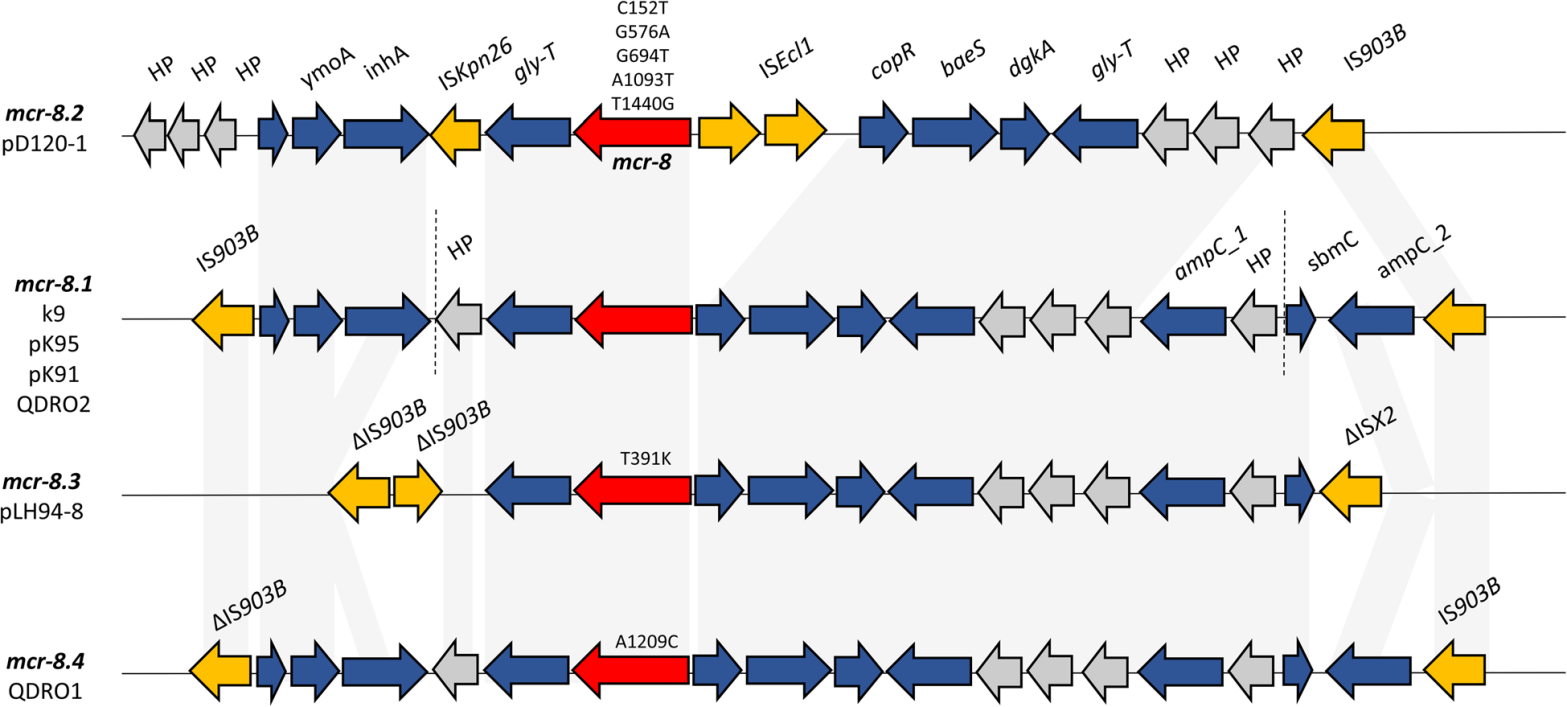
Schematic representation of the genetic environments of various *mcr-8* variants. *mcr-8* is shown in red. Amino acid substitutions compared to *mcr-8.1* are shown above each arrow. Hypothetical proteins (HP) are shown in grey and insertion sequences in yellow. Dotted vertical lines indicate the contig boundaries carrying *mcr-8.1* in k9

The genetic environment of *mcr-8.1* recovered from k9 was further compared with other *mcr-8* variants. The amino acid sequences and the genetic environment of *mcr-8.1* was most similar to *mcr-8.3* and *mcr-8.4* then to *mcr-8.2*, the latter being recovered from *K. quasipneumoniae* in China [28]. *mcr-8.2* also harboured more mutations compared to *mcr-8.1* than the other variants (Fig. 1).

**Fig. 2:**
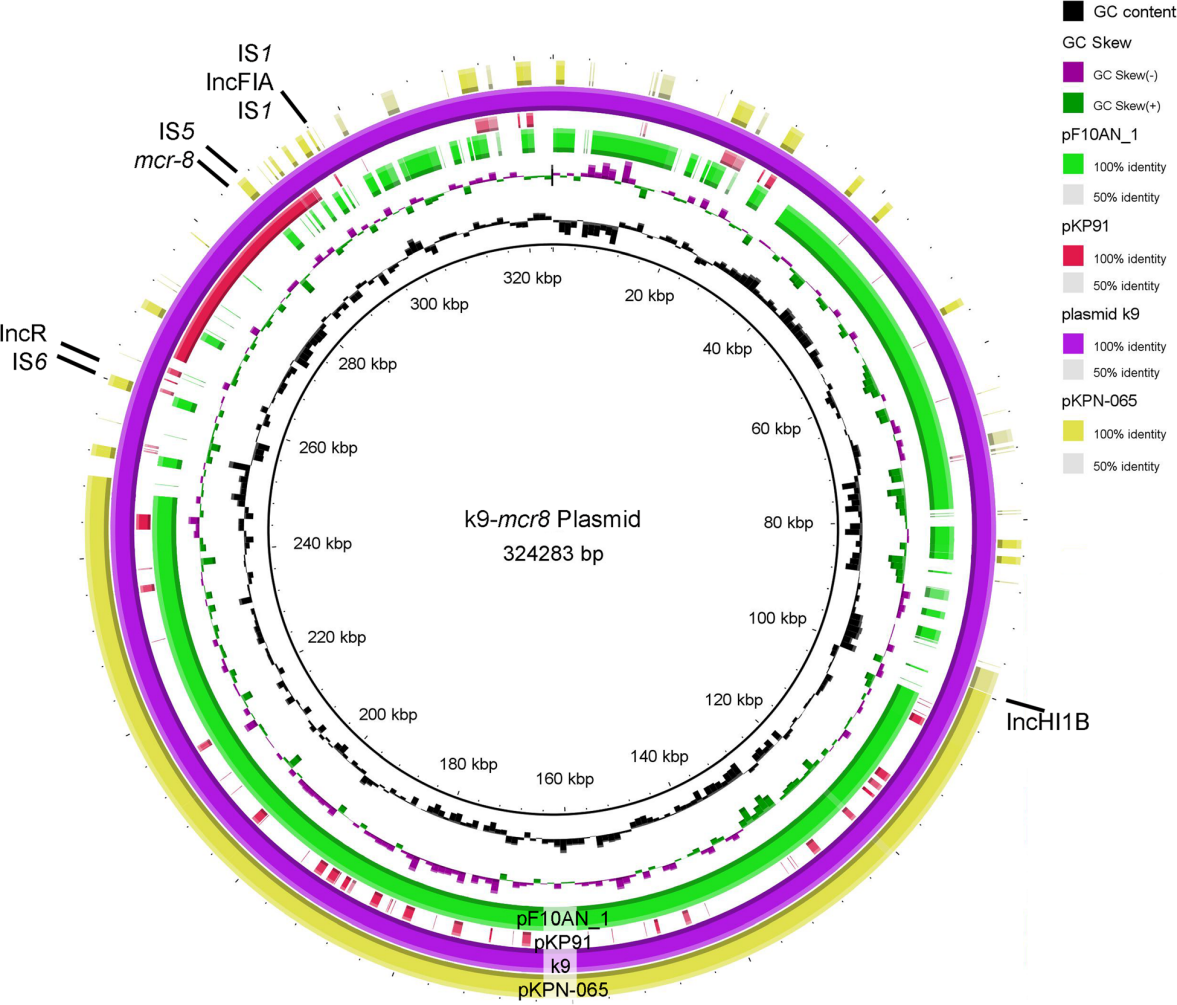
Circular graphical map of *mcr-8.1* plasmid in k9. The multireplicon plasmid in k9 was compared to pF10AN_1, AR_0158 plasmid, pKP91, pKPN-065, pR50–74, pYDC676 and pKP91 plasmids. Circles indicate, from inside to outwards: GC skew; GC content; Blast hits to various plasmids used as references. *Rep* genes and *mcr-8* locations are indicated on the figure. Inc. groups are shown in bold

*mcr-8.1* was located on a 324,283 bp multireplicon conjugative plasmid pk9 showing highest nucleotide similarity of 99–100% to plasmids: pF10AN_1 (305,552 bp; query coverage = 79.46%; Accession no. CP026154.1) and pKPN-065 (170,926 bp; query coverage = 90.16%; Accession no. CP015026.1) isolated from China and USA [29], respectively. The multireplicon plasmid harboured IncR and IncFIA *rep* genes also found in pKP91 and IncHI1B *rep* gene also found in pKPN-065. IncFIA was bracketed by two IS*1* insertion sequences while IS*6* was present downstream of IncR. Parts of pk9 plasmid carrying various AMR determinants also aligned to pR50–74 (Accession no. CP040362.1) collected from a rabbit fecal sample in China in 2017, pYDC676 collected from patients in the USA in 2014 (Accession no. KT225462.1) [30] and pAR_0158 collected in 2017 (Accession no. CP021699) (Fig. 2).

A plethora of resistance genes were detected on pk9. An AMR cassette was located between 14,200 bp and 18,300 bp encoding *sul1*, *aadA16*, *dfrA27*, *arr-3* and *aac(6′)-Ib-cr5*. It closest identity (99.93% identity, query coverage: 86%) to *Enterobacter hormaechei* plasmid pM206-NDM1 collected in Japan (Accession no. AP018830.1) [31]. Another similar cassette was located between 301,000 bp and 310,200 bp encoding *sul1*, *qnr*, *sul1*, *aadA16* and *aac(6′)-Ib-cr5* showing 100% identity and 98% coverage to *K. pneumoniae* Kp_29407 (Accession no. LR736030.1). Other resistance determinants present on the pk9 included *tetA, qnrB4, bla*_DHA-1_*, apha1b, sul3, aadA1–3, cmlA, floR* and *ampC*. Various sulfonamide resistance genes were identified including four copies of *sul1* in addition to *sul3* and *bcr* (bicyclomycin resistance protein).

## Discussion

The plasmid-mediated spread of *mcr* gene variants represents a major health concern worldwide. In this study, we identified a multireplicon plasmid carrying the *mcr-8.1* gene variant in a *K. pneumoniae* clinical isolate recovered from a urine sample in Lebanon. This represents the first report of *mcr-8* from Lebanon. *mcr-8.1* showed 100% gene sequence similarity to *mcr-8.1* encoded by pKP91 plasmid originating from a *K. pneumoniae* KP91 collected in 2018 from swine fecal material in China [4]. The detection of colistin resistance determinants in both humans and animals directly supports the One Health perspectives that focus on the spread of drug resistance between the environment, humans and animals [31, 32]. Since 2016, nine different plasmid-mediated *mcr* variants have been described (*mcr-1* to *mcr-9*) from human, animal and environmental sources [2–4], showing the prevalence of the *mcr* gene in the wildlife and particularly in surface water samples [33].

*mcr-8.1* was located on a 324,283 bp multireplicon plasmid showing highest nucleotide similarity (99–100%) to plasmids pF10AN_1 (Accession no. CP026154.1) and pKPN-065 (Accession no. CP015026.1) isolated from China and USA [31], respectively. The multireplicon plasmid harboured IncR and IncFIA *rep* genes also found in pKP91 [4] and IncHI1B *rep* gene also found in pKPN-065. Recently, *mcr-8.2* was detected on a large, hybrid plasmid containing IncQ, IncR, and IncFII replicons [34] confirming the notion that hybrid multi-replicon plasmids are emerging as vehicles for *mcr* gene spread [34].

Colistin resistant bacteria and the *mcr* gene family could be transmitted via the food chain, and so its prudent use in both human and veterinary medicine is of paramount importance [35]. Polymyxins were classified as the “Highest Priority Critically Important Antimicrobials” by the World Health Organization (WHO) because of the increasing usage of colistin to treat serious infections in humans in many parts of the world [35]. Further surveillance and screening for *mcr-8* in livestock and animal farms in Lebanon as well as in imported meat and poultry is recommended to track the environmental influences contributing to the development and dissemination of resistance determinants propagating on large mobile genetic vehicles.

## Supplementary information

**Additional file 1: Table S1.** Antimicrobial susceptibility and MIC results of *K. pneumoniae* k9. Antimicrobial susceptibility was performed using the disk diffusion technique; MICs of ertapenem, imipenem and meropenem were determined using the E-test methodology; MIC of colistin was determined using the broth microdilution method. Antibiotics were divided by their drug categories. GM: gentamycin 10μg; IP: imipenem; MEM: meropenem 10μg; IPM: imipenem 10μg; PM: cefepime; CTX: cefotaxime 30μg; CXM: cefuroxime 30μg; FEP: cefepime 30μg; CZD: ceftazidime 10μg; CZN: cefazolin 30μg; SCF: cefoperazone/sulbactam 75/30μg; FOX: cefoxitin 30μg; NOR: norfloxacin 5μg; CIP: ciprofloxacin 5μg; FAD: fusidic acid 10μg; TGC: tigecycline 15μg; CMN: clindamycin 2μg; ERY: erythromycin 15μg; ATM: aztreonam 30μg; LZD: linezolid 30μg; AMX: amoxicilin 25μg; PIR: pipercacillin 100μg; AMP: amipicillin 10μg; TZP: pipercacillin/tazobactam 100/10μg; FOS: fosfomycin 200μg; TE: tetracycline 30μg; TS: trimethoprim/sulfamethoxazole; CO: colistin; Dark blue: resistant, light blue: intermediate resistance, white: susceptible; MICs are in μg/mL; NA: not available.

**Additional file 2: Figure S1.** SNAP plots with potential synonymous and non-synonymous substitutions in *pmrA* (A) and *pmrB* (B). Alignment was performed against the query sequence of *K. pneumoniae* MGH 78578 wild-type chromosomal genes.

## Data Availability

This Whole Genome Shotgun project has been deposited at DDBJ/ENA/GenBank under the accession WTCT00000000. The version described in this paper is version WTCT01000000. The pk9 plasmid sequence was submitted to NCBI on 17/02/2020 under the submission number SUB6977004.

## Abbreviations

CRKP: Carbapenem resistant *Klebsiella pneumoniae*
MALDI-TOF: Matrix-Assisted Laser Desorption/Ionization Time of Flight
AUBMC: American University of Beirut Medical Centre
CLSI: Clinical & laboratory standards institute
MIC: Minimal inhibitory concentration
EUCAST: European Committee on Antimicrobial Susceptibility Testing
PBRT: Plasmid based replicon typing
PCR: Polymerase chain reaction
BRIG: Blast Ring Image Generator
AMR: Antimicrobial Resistance
XDR: Extensively drug resistant
WHO: World Health Organization
