# Supplementary material for: First report of plasmid-mediated colistin resistance mcr-8.1 gene from a clinical Klebsiella pneumoniae isolate from Lebanon
Source: Antimicrob Resist Infect Control. 2020 Jun 26;9:94. doi: 10.1186/s13756-020-00759-w (PMC7318401; doi:10.1186/s13756-020-00759-w)
Supplement: Supplementary file 1 — Additional file 1: Table S1. Antimicrobial susceptibility and MIC results of K. pneumoniae k9. Antimicrobial susceptibility was performed using the disk diffusion technique; MICs of ertapenem, imipenem and meropenem were determined using the E-test methodology; MIC of colistin was determined using the broth microdilution method. Antibiotics were divided by their drug categories. GM: gentamycin 10μg; IP: imipenem; MEM: meropenem 10μg; IPM: imipenem 10μg; PM: cefepime; CTX: cefotaxime 30μg; CXM: cefuroxime 30μg; FEP: cefepime 30μg; CZD: ceftazidime 10μg; CZN: cefazolin 30μg; SCF: cefoperazone/sulbactam 75/30μg; FOX: cefoxitin 30μg; NOR: norfloxacin 5μg; CIP: ciprofloxacin 5μg; FAD: fusidic acid 10μg; TGC: tigecycline 15μg; CMN: clindamycin 2μg; ERY: erythromycin 15μg; ATM: aztreonam 30μg; LZD: linezolid 30μg; AMX: amoxicilin 25μg; PIR: pipercacillin 100μg; AMP: amipicillin 10μg; TZP: pipercacillin/tazobactam 100/10μg; FOS: fosfomycin 200μg; TE: tetracycline 30μg; TS: trimethoprim/sulfamethoxazole; CO: colistin; Dark blue: resistant, light blue: intermediate resistance, white: susceptible; MICs are in μg/mL; NA: not available. [file 13756_2020_759_MOESM1_ESM.pdf]

Table S1. Antimicrobial susceptibility and MIC results of *K. pneumoniae* k9. Antimicrobial susceptibility was performed using the disk diffusion technique; MICs of ertapenem, imipenem and meropenem were determined using the E-test methodology; MIC of colistin was determined using the broth microdilution method. Antibiotics were divided by their drug categories. GM: gentamycin 10ug; IP: imipenem; MEM: meropenem 10ug; IPM: imipenem 10ug; PM: cefepime; CTX: cefotaxime 30ug; CXM: cefuroxime 30ug; FEP: cefepime 30ug; CZD: ceftazidime 10ug; CZN: cefazolin 30ug; SCF: cefoperazone/sulbactam 75/30ug; FOX: cefoxitin 30ug; NOR: norfloxacin 5ug; CIP: ciprofloxacin 5ug; FAD: fusidic acid 10ug; TGC: tigecycline 15ug; CMN: clindamycin 2ug; ERY: erythromycin 15ug; ATM: aztreonam 30ug; LZD: linezolid 30ug; AMX: amoxicilin 25ug; PIR: pipercacillin 100ug; AMP: amipicillin 10ug; TZP: pipercacillin/tazobactam 100/10ug; FOS: fosfomycin 200ug; TE: tetracycline 30ug; TS: trimethoprim/sulfamethoxazole; CO: colistin; Dark blue: resistant, light blue: intermediate resistance, white: susceptible; MICs are in µg/mL; NA: not available.

	**Category**	**Aminoglycosides**	**Carbapenems**			**Cephalosporin**						**Cephamycin**	**Fluoroquinolones**		**Fusidane**	**Glycyclines**	**Lincosamide**	**Macrolide**	**Monobactam**	**Oxazolidinones**	**Penicillin**			**β-lactam combination**	**Phophonic Acids**	**Tetracycline**	**Folate Pathway Inhibitors**	**Polymyxins**
**Abbreviation**	**Isolate**	GM	IP	MEM	IPM	PM	CTX	CXM	FEP	CZD	CZN	SCF	FOX	NOR	CIP	FAD	TGC	CMN	ERY	ATM	LZD	AMX	PIR	AMP	TZP	FOS	TE	TS	CO
**Disk Diffusion**	*K. pneumoniae* K9					NA																						NA	NA
**MICs**			0.25	0.25	0.016	3.5									>32		2											>32	10
	E. coli J53 Transconjugant		0.25	0.002	0.012	0.032									0.016		0.125											0.032	10
