# Supplementary figures and images for: First report of plasmid-mediated colistin resistance mcr-8.1 gene from a clinical Klebsiella pneumoniae isolate from Lebanon
Source: Antimicrob Resist Infect Control. 2020 Jun 26;9:94. doi: 10.1186/s13756-020-00759-w (PMC7318401; doi:10.1186/s13756-020-00759-w)

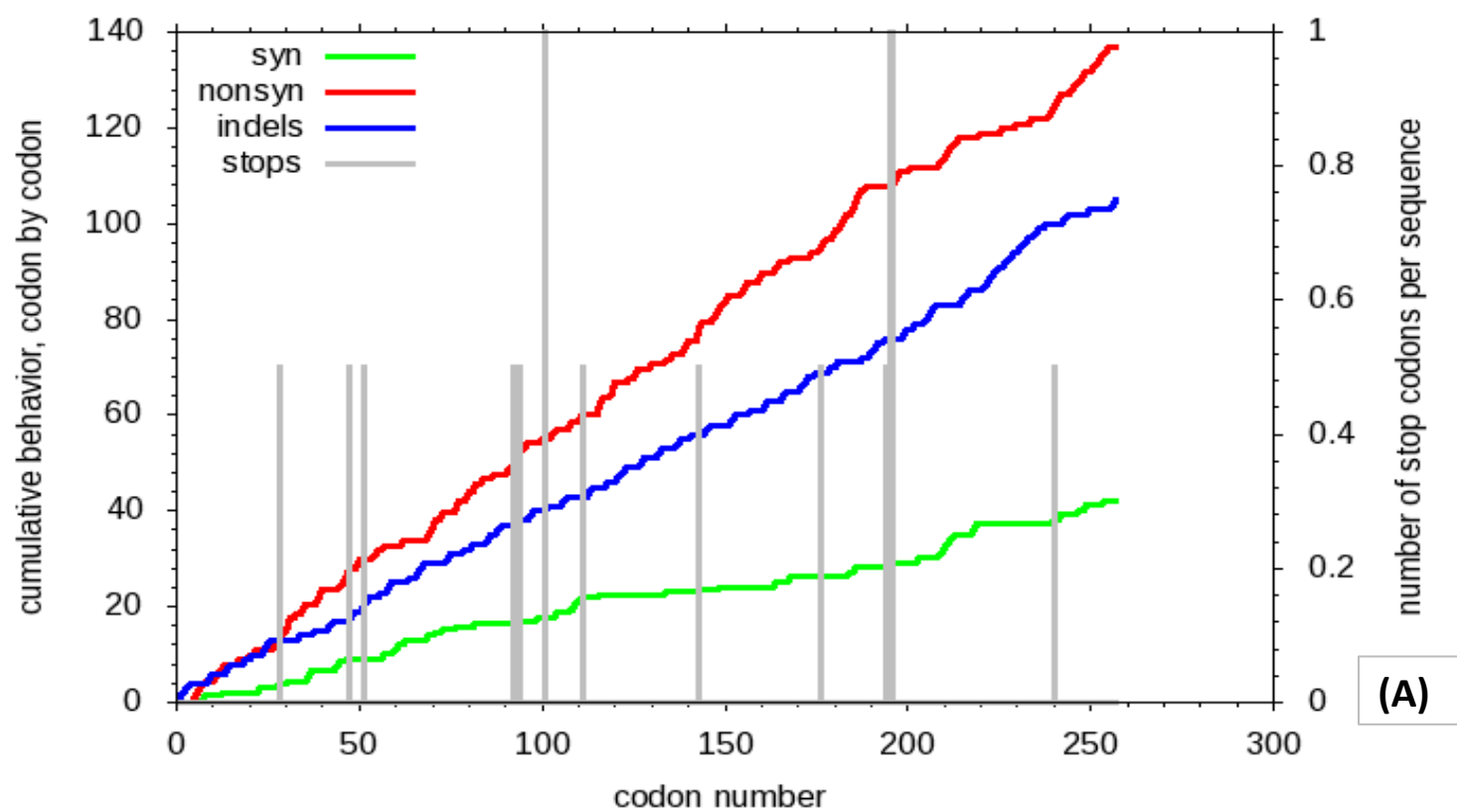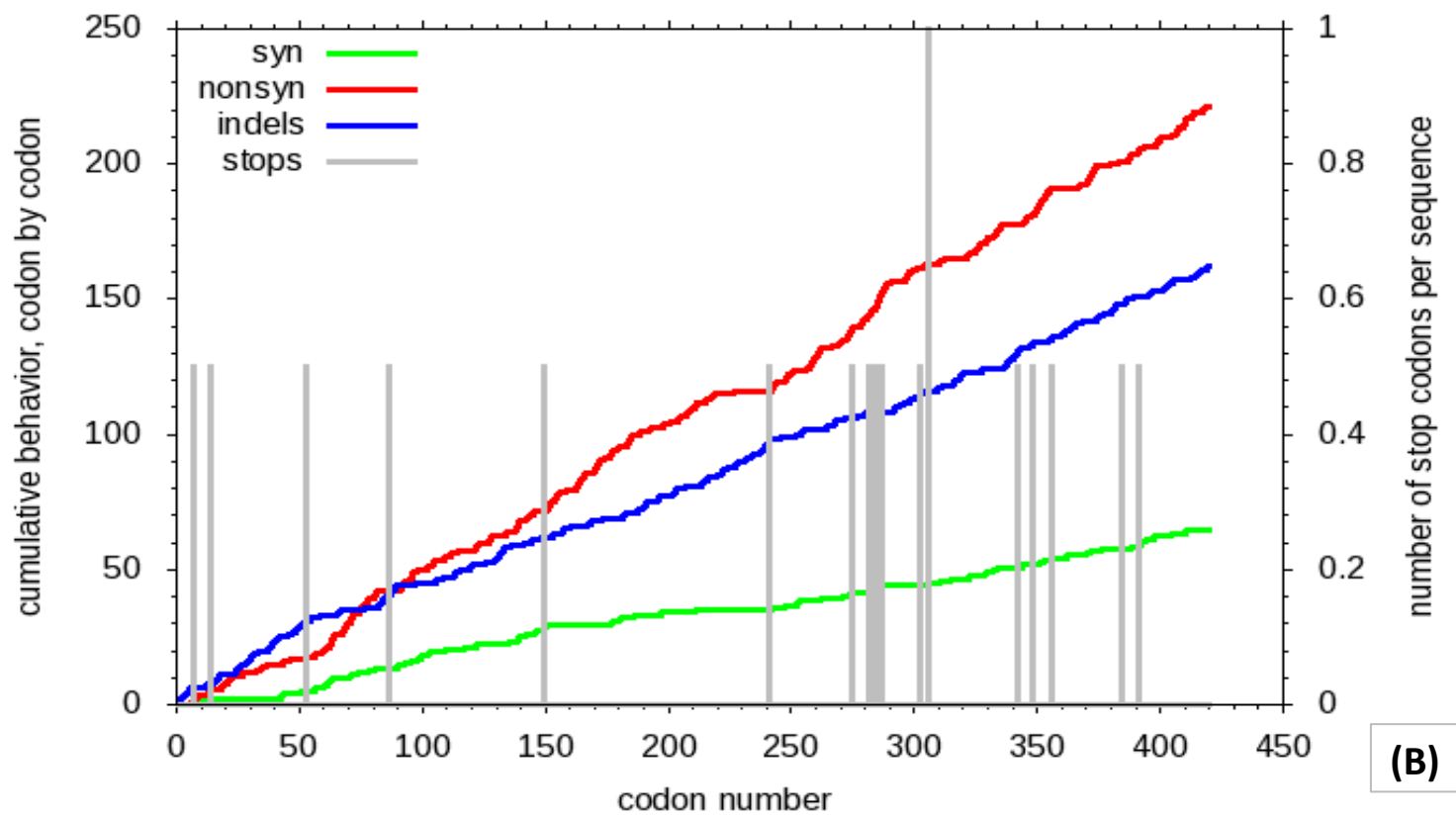

Supplement: Supplementary file 2 — Additional file 2: Figure S1. SNAP plots with potential synonymous and non-synonymous substitutions in pmrA (A) and pmrB (B). Alignment was performed against the query sequence of K. pneumoniae MGH 78578 wild-type chromosomal genes. [file 13756_2020_759_MOESM2_ESM.pdf]
